# Antioxidant Defenses Confer Resistance to High Dose Melphalan in Multiple Myeloma Cells

**DOI:** 10.3390/cancers11040439

**Published:** 2019-03-28

**Authors:** Claire Gourzones, Céline Bellanger, Sylvain Lamure, Ouissem Karmous Gadacha, Elvira Garcia De Paco, Laure Vincent, Guillaume Cartron, Bernard Klein, Jérôme Moreaux

**Affiliations:** 1 IGH, CNRS, University of Montpellier, 34000 Montpellier, France; cgourzones@gmail.com (C.G.); Celine.Bellanger@univ-nantes.fr (C.B.); ouissem.karmous-gadacha@igh.cnrs.fr (O.K.G.); elvira.garcia-de-paco@igh.cnrs.fr (E.G.D.P.); bernard.klein@inserm.fr (B.K.); 2 Department of Clinical Hematology, CHU Montpellier, 34395 Montpellier, France; s-lamure@chu-montpellier.fr (S.L.); l-vincent@chu-montpellier.fr (L.V.); g-cartron@chu-montpellier.fr (G.C.); 3 Univ Montpellier, UFR de Médecine, 34000 Montpellier, France; 4 Univ Montpellier, UMR CNRS 5235, 34000 Montpellier, France; 5 Department of Biological Hematology, CHU Montpellier, 34295 Montpellier, France

**Keywords:** GSH, multiple myeloma, reactive oxygen species, melphalan, drug resistance, NRF2

## Abstract

Background: Multiple myeloma (MM) is the second most common hematological cancer after lymphoma. It is characterized by the accumulation of clonal malignant plasma cells within the bone marrow. The development of drug resistance remains a major problem for effective treatment of MM. Understand the mechanisms underlying drug resistance in MM is a focal point to improve MM treatment. Methods: In the current study, we analyzed further the role of redox imbalance induction in melphalan-induced toxicity both in human myeloma cell lines (HMCLs) and primary myeloma cells from patients. Results: We developed an in-vitro model of short-term resistance to high-dose melphalan and identified that pretreatment with physiological concentration of GSH protects HMCLs from melphalan-induced cell cycle arrest and cytotoxicity. We validated these results using primary MM cells from patients co-cultured with their bone marrow microenvironment. GSH did not affect the ability of melphalan to induce DNA damages in MM cells. Interestingly, melphalan induced reactive oxygen species, a significant decrease in GSH concentration, protein and lipd oxydation together with NRF2 (NF-E2-related factor 2) pathway activation. Conclusions: Our data demonstrate that antioxidant defenses confers resistance to high dose melphalan in MM cells, supporting that redox status in MM cells could be determinant for patients’ response to melphalan.

## 1. Introduction

Multiple myeloma (MM) is a malignant plasma cell disorder affecting approximately 70,000 new patients/year around the world. Treatment of this disease is regularly improving with the development of new drugs and the design of better combinations [1]. Current treatment involves four major categories of drugs: proteasome inhibitors, immunomodulatory drugs, the alkylating agent melphalan, and high dose dexamethasone. Despite the advances in treatment and increase in overall survival, a majority of patients relapse and ultimately die. In parallel to the design of new drugs, it is therefore critical to understand why tumor cells and/or the tumor environment eventually become resistant to the various drugs and patient treatment fails.

Melphalan is administered at low concentrations for initial therapy of patients not eligible for autologous stem-cell transplantation (ASCT) and is, at high concentration, the most common conditioning treatment for patients undergoing ASCT [2]. The current view of melphalan activity in MM is through its DNA genotoxicity [3]. Indeed, melphalan binds guanosine and forms monoadducts (95% of melphalan molecules) and interstrand cross links (ICLs, 5%) [4]. These melphalan DNA adducts impair RNA and DNA polymerases progression and need to be eliminated by various DNA repair pathways to restore cell function [5]. The removal of melphalan adducts creates DNA breaks, which need to be further fixed by DNA repair pathways [5,6]. Alkylating agents bind to DNA but it is important to emphasize that 75–85% of these molecules bind also to proteins and could interfere with their activity [7,8].

Several mechanisms of resistance to melphalan have been described in MM cells (MMCs) such as an increased ability to repair melphalan-induced ICLs and DNA breaks [3,9], mainly through the base excision repair pathway and Fanconi anemia DNA repair pathways [6,10,11,12,13]. Other studies have shown a decreased uptake of melphalan through down-regulation of the L-type amino acid transporter 1 (LAT1 or SLC7A5) influx transporter [14] or an increased efflux through overexpression of the efflux transporter MDR1 [15]. RECQ1 helicase overexpression also protects MM cells from melphalan cytotoxicity in a subgroup of patients associated with a poor outcome [16].

Redox homeostasis is highly regulated within cells by a set of enzymatic and non-enzymatic components that regulate the level of reactive oxygen species (ROS), most of them produced by respiratory chain in mitochondria [17]. A major role of alkylating drugs in modifying redox potential and in particular of ROS levels has already been documented in various cancers [18,19]. Such a mechanism has been described for melphalan, which binds and reduces the activity of thioredoxin reductase increasing the production of reactive oxygen species [20]. A more recent study showed that melphalan and bendamustine induce ROS and p53 pathway activation in MM cells [21]. Pathways involved in reactive oxygen species scavenging could also play a role in resistance to melphalan. According to that, a 2-fold increase in intracellular non-protein thiols in the RPMI8226 human myeloma cell line (HMCL) induced resistance to melphalan after several months of treatment [22].

In the current study, we have explored further the role of redox imbalance induction in melphalan-induced toxicity both in HMCLs and primary myeloma cells. To this end we have investigated the effect of reduced glutathione (GSH) as well as other antioxidants and monitored induction of ROS and terminal markers of oxidative stress.

We showed in an in-vitro model of short-term resistance to melphalan that pretreatment with GSH protects HMCLs from melphalan-induced cell cycle arrest and cytotoxicity. We confirmed this observation in primary cells from patients and also with other antioxidants. Next, we demonstrated that melphalan induces reactive oxygen species, a decrease in GSH concentration and NRF2 (NF-E2-related factor 2) pathway activation. This study shows that increasing antioxidant defenses confers resistance to high dose melphalan in MM cells, supporting that redox status and especially antioxidant and GSH concentrations in MM cells could be determinant for patients’ response to melphalan.

## 2. Results

### 2.1. Antioxidants Protect Cells from Melphalan-Induced Toxicity

**Figure 1:**
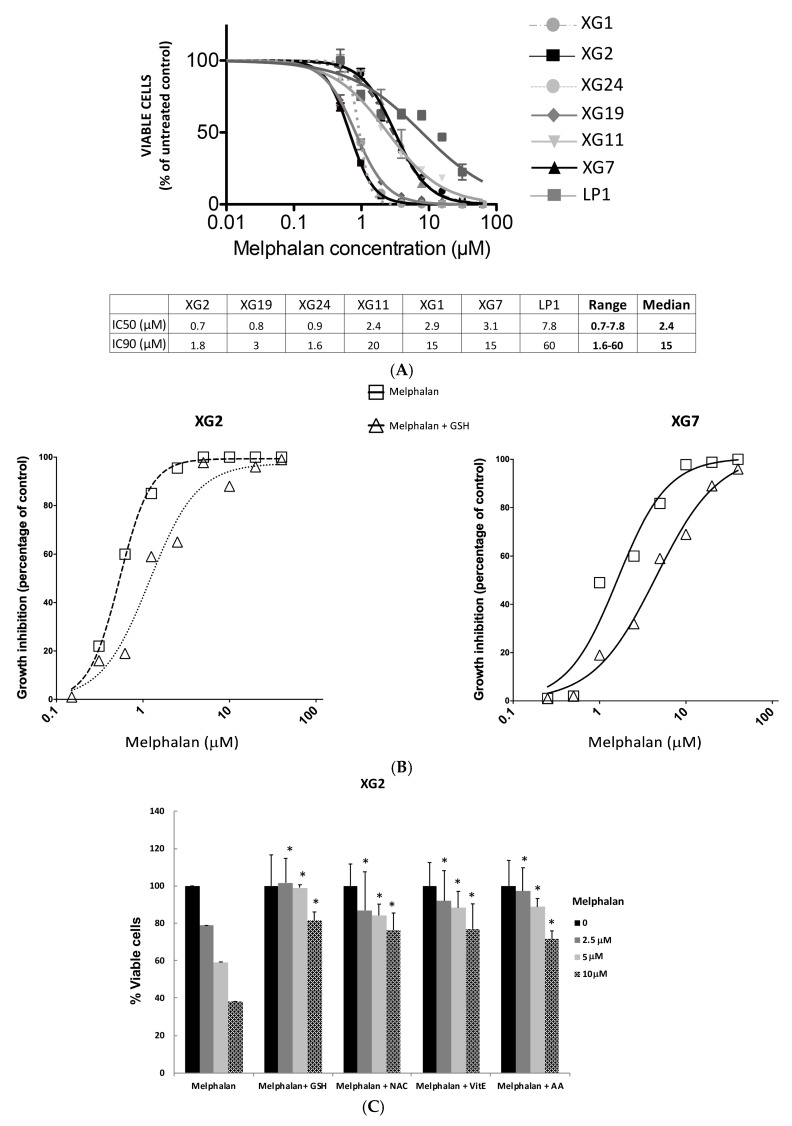
Antioxidants protects cells from melphalan-induced toxicity. (**A**) Melphalan-induced growth inhibition of 7 HMCLs. Seven HMCLs were treated with serial concentrations of melphalan and the percentage of viable cells compared to untreated control remaining 4 days after the onset of treatment was assessed by the ATP cell titer glo assay. Results are mean values ± standard deviation (SD) of 3 separate experiments. The inhibitory concentrations leading to a 50% (IC50) or 90% (IC90) decrease in viable cells compared to untreated cells at day 4 are indicated for each cell line in the table below the figure. (**B**) Addition of 5 mM of GSH protects MM cells from Melphalan induced toxicity. XG2 and XG7 HMCLs were cultured for 96 h in 96-well flat-bottom microtitre plates in RPMI 1640 medium, 10% fetal calf serum, 2 ng/mL IL-6 culture medium (control) and graded concentration of Melphalan with or without GSH (5 mM). Data are mean values of three experiments determined on sextuplet culture wells. (**C**) XG2 HMCL was cultured for 24 h in 96-well flat-bottom microtitre plates in RPMI 1640 medium, 10% fetal calf serum, 2 ng/mL IL-6 culture medium (control) and graded concentration of Melphalan with or without GSH (5 mM), NAC (5 mM), AA (100 μM) or Vit E (50 μM). Data are mean values ± SD of three experiments determined on sextuplet culture wells. * *p* < 0.05 compared to melphalan alone using a Wilcoxon test for pairs.

To determine the range of melphalan concentrations inducing MM cell death and the differences of sensitivity between cell lines, dose response-curves were performed using seven HMCLs representative of the molecular heterogeneity encountered in MM patients [23] (Figure 1A). The 50% inhibitory concentration (IC50) varied 11-fold (median = 2.4 µM; range: 0.7 to 7.8 µM) among the seven HMCLs investigated and the 90% inhibitory concentrations (IC90) 37.5-fold (median: 15 µM; range: 1.6 to 60 µM) (Figure 1A). For the following experiments, we selected XG2 (*TP53*^−/mut^ HMCL), the most sensitive cell line among those tested, and XG7 HMCL (*TP53* wild type) with a 8-fold higher IC90 for melphalan treatment. In order to define a role of ROS induction in melphalan-induced cytoxicity, MM cells were treated by melphalan alone or in combination with various antioxidants (Figure 1B,C). 5 mM of GSH protected myeloma cells from melphalan-induced mortality (Figure 1B and Figures S5 and S6). The same protective effect was observed in XG2 cell line pretreated with N-acetylcysteine (NAC), vitamin E and ascorbic acid (AA) (Figure 1C) (*p* < 0.05). Furthermore, the level of intracellular GSH was 1.4 higher in XG7 compared to XG2 HMCL (Figure S1).

### 2.2. Addition of GSH Prevents Cell Cycle Arrest after Treatment with Melphalan and Increases Resistance to High Dose Melphalan

**Figure 2:**
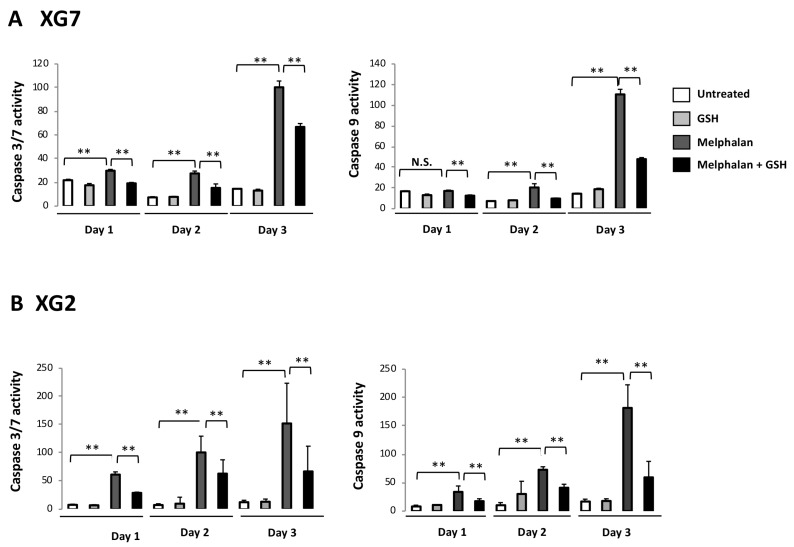
GSH inhibits caspase induction after treatment with melphalan. Induction of caspases was monitored after treatment with melphalan with a luciferase assay. (**A**) Activation of caspases after treatment of XG7 cells. XG7 cells were treated with 15 µM of melphalan with or without cotreatment with GSH (5 mM) and induction of caspases was evaluated at day 1, 2 and 3 post-treatment with a luciferase assay. Graphs shows results of bioluminescence intensity for caspase assay. Bioluminescence intensity resulting from the addition of Cell titer glo assay reagent and measuring ATP concentration and therefore reflecting the count of viable cells was used for normalization. Results for activation of caspase 3/7 are shown in the left panel and for caspase 9 in the right panel. (**B**) caspase activation after treatment of XG2 cell line. Same experiment after treatment of XG2 cells with 5 µM melphalan. ** *p* < 0.05 using a Wilcoxon test for pairs.

To mimic treatment of patients with high dose melphalan (HDM), we designed a short-term resistance model: HMCLs were treated with concentrations equal or above their IC90, which are within the range of the peak concentrations reached in plasma of patients treated with high dose melphalan [24] (Figure S2). On day 2 post-treatment with 15 µM (IC_90_) melphalan, wild-type *TP53* XG7 myeloma cells presented a decreased BrdU incorporation and accumulated in the G2 phase (Figure S2A,B). They started to die 24 h post treatment and most surviving cells remained blocked in the G2/M phases of the cell cycle at day 2. Apoptosis was evidenced by effector caspase 3/7 activation at day 1 (*p* < 0.01) and caspase 9 activation at day 2 (*p* < 0.01) (Figure 2A). At day 6, the vast majority of myeloma cells were dead, except a small percentage (4%), which resumed full cell cycling and cell expansion. Therefore, small fraction of XG7 MM cells show intrinsic resistance to the melphalan IC_90_ (15 µM), with a rapid cell cycling recovery. The same observation was made with the *TP53*^−/mut^ XG2 HMCL exposed to its IC97 of melphalan (i.e., 5 µM) (Figure S2C,D and Figure 2B). Only 10% XG2 MM cells survived to high dose melphalan at day 4, but rapidly resumed cell cycling and further expansion.

**Figure 3:**
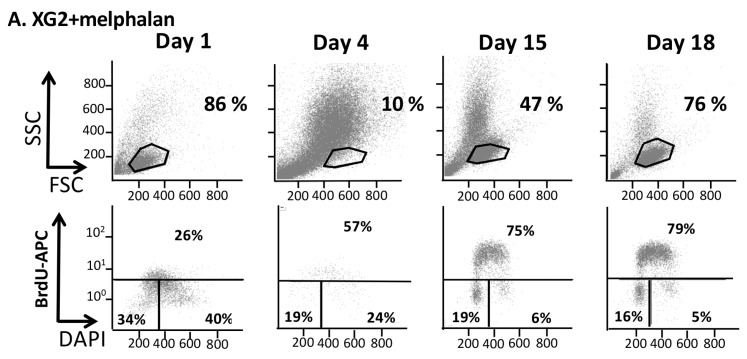

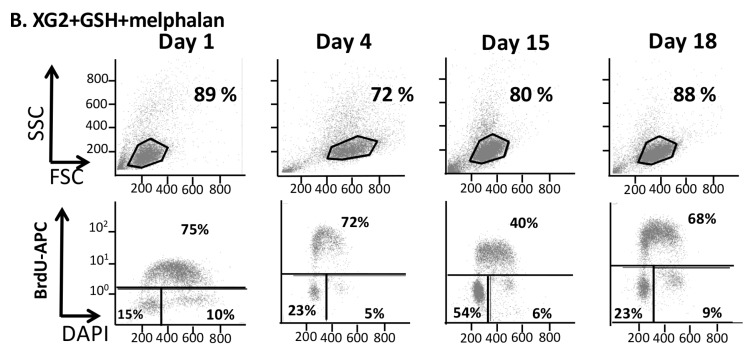
Kinetics of melphalan effect on the viability and cell cycle of XG2 myeloma cells. (**A**) XG2 cells were treated with 5 µM melphalan and FSC/SSC and cell cycle analyses were performed by flow cytometry, using BrdU incorporation and labeling with an anti-BrdU antibody and DAPI, before treatment (day 0) and at day 1, 4, 15, 18 after the onset of treatment. (**B**) The percentage of cells in each phase of the cell cycle is indicated. The percentage of viable cells was determined using FSC/SSC characteristics and cell count.

We next sought to better characterize the protective effect of GSH in this model of treatment with high melphalan concentration. 5 mM of GSH promoted survival of XG2 or XG7 MM cells in our model. Data of one experiment representative of 3 for XG2 HMCL are shown in Figure 3. Four days after melphalan treatment, GSH addition resulted in a 4-fold increase in XG2 HMCL survival compared to control (*p* < 0.05, Table S1). These results were validated using XG7 HMCL (Figure S3). Surviving cells rapidly resume cell cycling and growth (Figure 3 and Figure S3). Treatment with 5 mM GSH significantly decreased caspase activation both in XG2 and XG7 cells (Figure 2).

### 2.3. GSH Efficiently Blocks Melphalan Induced Cell Death in Primary Myeloma Cells of Patients

**Figure 4:**
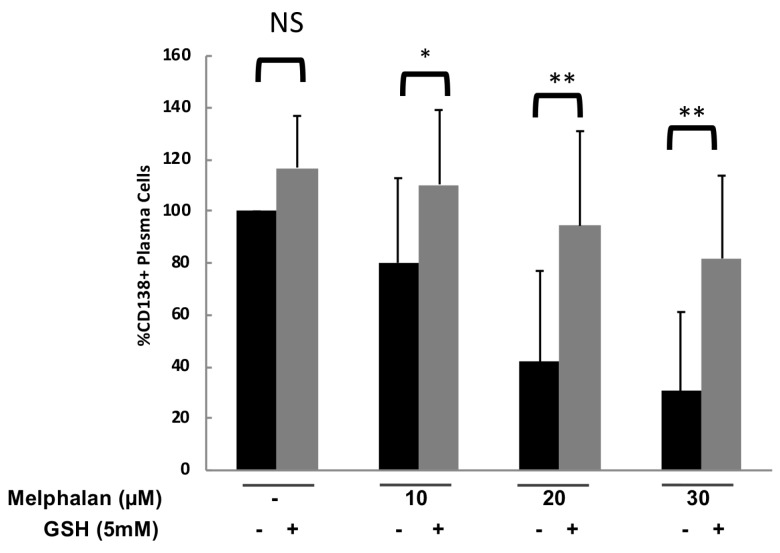
GSH protects CD138^+^ cells from melphalan-induced toxicity. Mononuclear cells from six different patients with MM were treated with 10, 20 and 30 µM melphalan with or without a cotreatment with 5 mM GSH. At day 4 of culture, the viability and total cell counts were assessed, for the 6 different samples, and the percentage CD138^+^ viable plasma cells was determined by flow cytometry. Results are median values of the numbers of myeloma cells in the culture wells normalized to the control. Results were compared with a Wilcoxon test for pairs. * *p* < 0.05; ** *p* < 0.005.

To confirm the protective effect of antioxidant compounds in primary myeloma cells from patients, bone marrow samples from 6 MM patients were treated with 5 mM GSH with or without 10, 20 or 30 µM melphalan. GSH treatment protected CD138^+^ tumor cells from melphalan-induced toxicity in all the patients investigated with a median 2.3 fold increase of the number of CD138^+^ cells (Figure 4) (*p* < 0.05, < 0.005 and < 0.005 for 10, 20 and 30 μM of melphalan, respectively).

### 2.4. Glutathione Did Not Affect the Ability of Melphalan to Induce DNA Lesions in MM Cells

**Figure 5:**
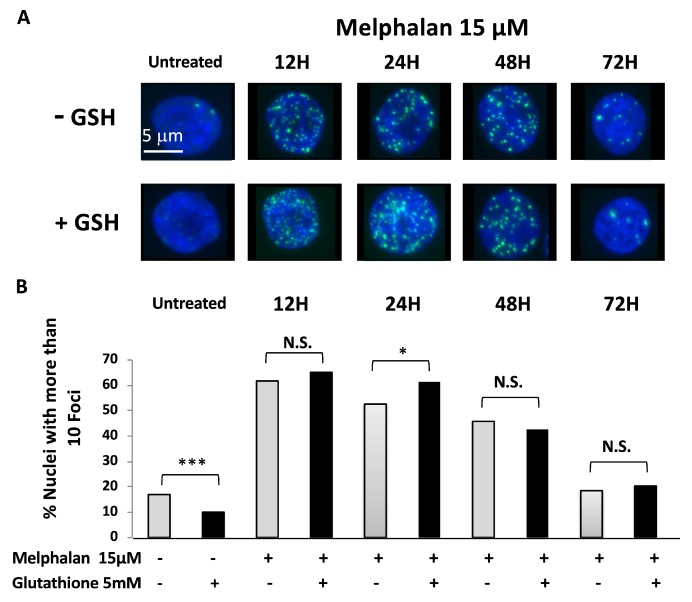
Pretreatment with GSH doesn’t prevent the induction of 53BP1 foci after treatment MM cells with melphalan. XG7 cells were treated with 15 µM of melphalan with or without a pretreatment with 5 mM GSH. Cells were harvested after 12, 24, 48 and 72 h, and 53BP1 foci detected using immunofluorescence (**A**). A representative image for each condition and the number of foci per nuclei are shown on the figure (**B**). N.S.: Not significant; * *p* < 0.05; *** *p* < 0.001.

A possible mechanism for the protective effect of GSH towards melphalan is an increase of melphalan metabolizing by glutathione S transferases, resulting in impairment of melphalan genotoxicity. GSH addition did not prevent induction of DNA damages by melphalan in XG7 and XG2 HMCLs (Figure 5A,B and Figure S4, respectively). Twelve and 24 h after IC_90_ melphalan treatment, the nuclei of more than 50% of XG7 myeloma cells had 10 or more DNA repair foci evidenced using anti-53BP1 antibodies. 53BP1 foci number detected after melphalan exposure was not significantly altered by GSH treatment (5 mM) (Figure 5A,B). Foci number decreased after 24 h suggesting repair of the DNA lesions.

### 2.5. Melphalan Treatment Induces ROS Production, which are Involved in Melphalan-Induced Cytotoxicity and Prevented by Addition of GSH

**Figure 6:**
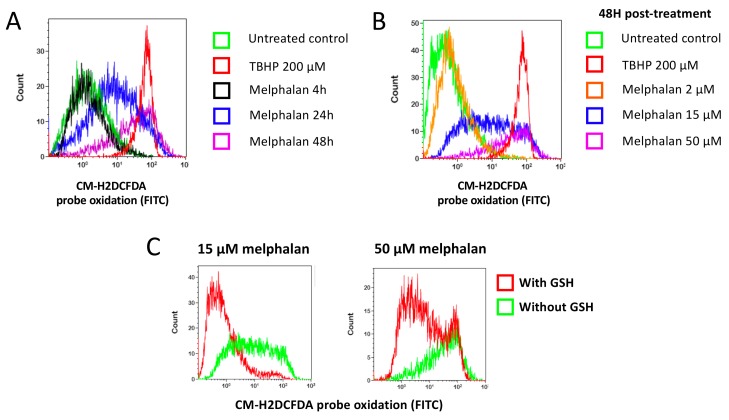
Detection of melphalan induced ROS in MM cells. (**A**) Kinetics of ROS induction after treatment with melphalan. XG7 cells were treated with 50 µM melphalan or tert-Butyl hydroperoxide (TBHP), a compound that induces oxidative stress, as a positive control. ROS were detected 4 h, 24 h or 48 h after the onset of treatment by the addition of the CM-H2DCFDA probe that becomes fluorescent when oxidized. Fluorescence was analyzed by flow cytometry. (**B**) Dose-dependent induction of ROS after treatment with melphalan. XG7 cells were treated with 2 µM melphalan (i.e., IC10), 15 µM melphalan (i.e., IC_90_) or 50 µM melphalan. TBHP was used as a positive control. (**C**) Prevention of ROS induction after treatment with melphalan by GSH addition. XG7 MM cells were treated with 15 µM melphalan or 50 µM melphalan with or without a pre-treatment with 5 mM GSH. The induction of ROS was tested 48 h after the onset of treatment with the detection of the CM-DCFDA probe oxidation by flow cytometry.

Glutathione is the major redox molecule in cells. In particular it scavenges ROS, preventing their accumulation and toxicity [17,25]. We hypothesized that GSH protected cells towards melphalan by scavenging ROS that may be induced after treatment with melphalan. To test our hypothesis, we analyzed ROS levels in HMCLs after treatment with different concentrations of melphalan and at different time points (Figure 6A,B). Melphalan increased ROS levels in myeloma cells using the CM-H2DCFDA probe, which is oxidized by hydrogen peroxide and hydroxyl radicals. ROS started to be detected 24 h after treatment with melphalan IC_90_ and increased for the first 48 h (Figure 6A). Later than 48 h, cells massively died preventing accurate ROS detection by FACS. ROS production became detectable with melphalan concentrations starting to induce myeloma cell killing and increased with rising melphalan concentrations (Figure 6B). tert-Butylhydroperoxide (TBHP), an organic peroxide and a well-known oxidative stress inducer was used as a positive control [26]. ROS induction after treatment with melphalan was prevented by a pretreatment with 5 mM GSH (Figure 6C).

### 2.6. Melphalan Induces GSH Depletion Together with Protein and Lipid Oxidation

**Figure 7:**
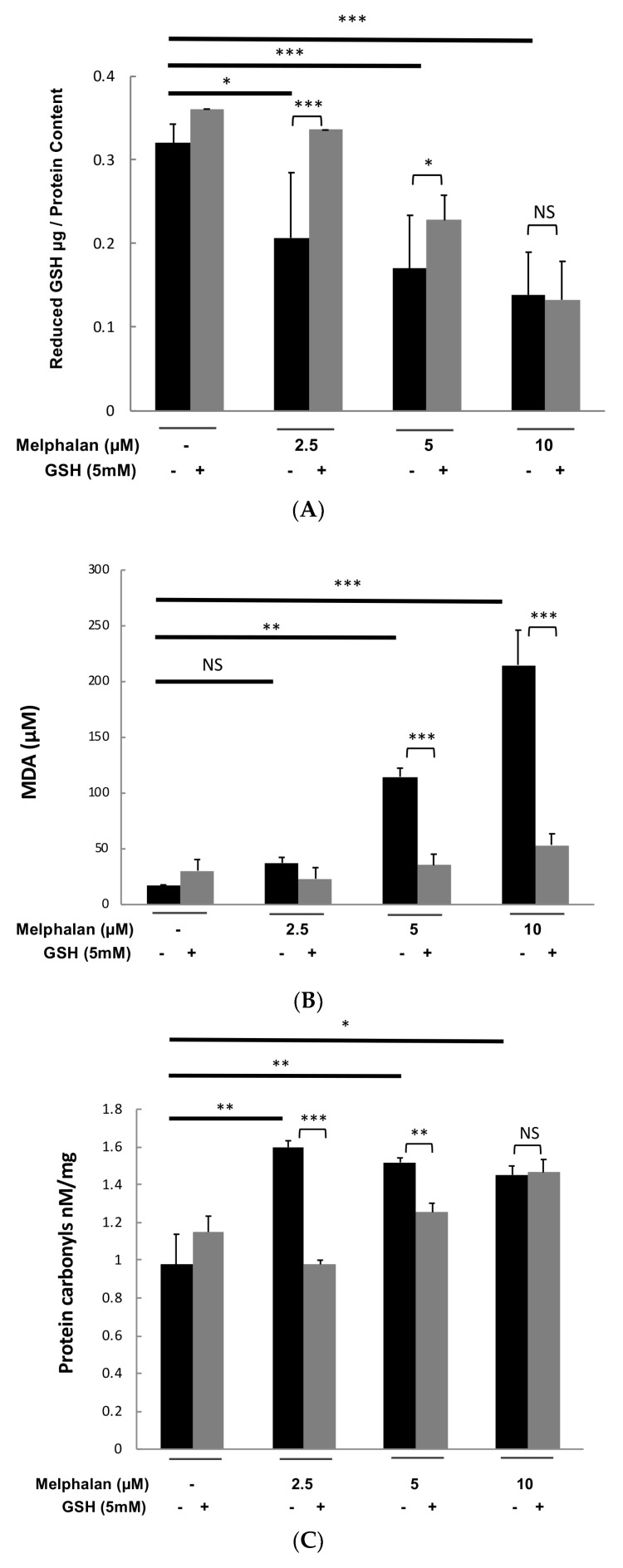

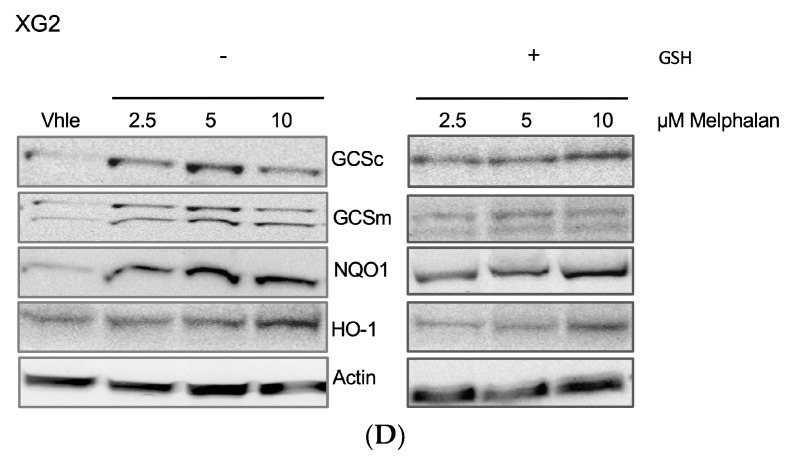
Melphalan induces GSH depletion together with protein and lipid oxidation. (**A**) XG2 HMCL was cultured with 2.5, 5 and 10 µM of melphalan in presence or absence of GSH (5 mM) during 3 days and GSH pool was measured using a Gluathione Assay kit according to the supplier’s instructions. Results are representative of five independent experiments. (**B**) XG2 HMCL was cultured with 2.5, 5 and 10 µM of melphalan in presence or absence of GSH (5 mM) during 3 days and monitoring of lipid peroxidation was performed using OxiSelectTM TBARS Assay Kit according to manufacturer’s instructions. Results are representative of five independent experiments. (**C**) XG2 HMCL was cultured with 2.5, 5 and 10 µM of melphalan in presence or absence of GSH (5 mM) during 3 days and quantification of protein carbonyl derivatives was performed using the OxiSelect Protein Carbonyl ELISA Kit according to the manufacturer’s instructions. Results are representative of five independent experiments. (**D**) XG2 HMCL was cultured with 2.5, 5 and 10 µM of melphalan in presence or absence of GSH (5 mM) during 3 days and protein expression of NRF2 targets, including the regulatory subunits of glutamate cysteine ligase (GCSc (73 kDa) and GCSm (31 kDa)), heme oxygenase-1 (HO-1; 28 kDa) and NAD(P)H Quinone Oxidoreductase 1 (NQO1; 29 kDa), was investigated using western blot. *** *p* < 0.001; ** *p* < 0.005; * *p* < 0.05.

We investigated whether melphalan could decrease the concentration of intracellular glutathione, the major redox molecule in cells. Treatment with 2.5, 5 and 10 µM of melphalan reduced respectively by 36%, 47% and 57% intracellular reduced glutathione (GSH) in XG2 myeloma cell line (Figure 7A). Adding 5 mM GSH prior exposure to different melphalan doses partially rescued intracellular GSH depletion (Figure 7A) but it promoted the survival of a majority of MM cells.

To confirm ROS induction after treatment with melphalan, the consequences of oxidative stress induction in treated myeloma cells were also investigated [27,28,29]. As presented in Figure 7B, treatment of XG2 cells with 2.5, 5 and 10 µM of melphalan induced a significant lipid peroxidation monitored by malondialdehyde quantification. GSH addition completely abrogated lipid peroxidation induced by melphalan (Figure 7B). Furthermore, melphalan treatment resulted also in significant protein carbonylation known as a biomarker of oxidative stress [28,29] (Figure 7C). GSH treatment inhibited protein carbonylation induced by 2.5 and 5 µM melphalan treatment (Figure 7C).

NRF2 is a key element in the regulation of antioxidant defense. According to our previous results, melphalan treatment also activated NRF2 antioxidant pathway with a significant induction of downstream targets of NRF2 including heme oxygenase-1 (HO-1) [30], NAD(P)H Quinone Oxidoreductase 1 (NQO1) [31] and both the catalytic and the regulatory subunits of glutamate cysteine ligase (GCSc and GCSm) [32] (Figure 7D). Addition of GSH did not significantly affected the level of HO-1, NQO1, GCSc and GCSm after melphalan treatment. Altogether, these data demonstrated that melphalan treatment induces oxidative stress in MM cells.

### 2.7. Oxidative Stress Response Score is Associated with a Poor Outcome in MM Patients

**Figure 8:**
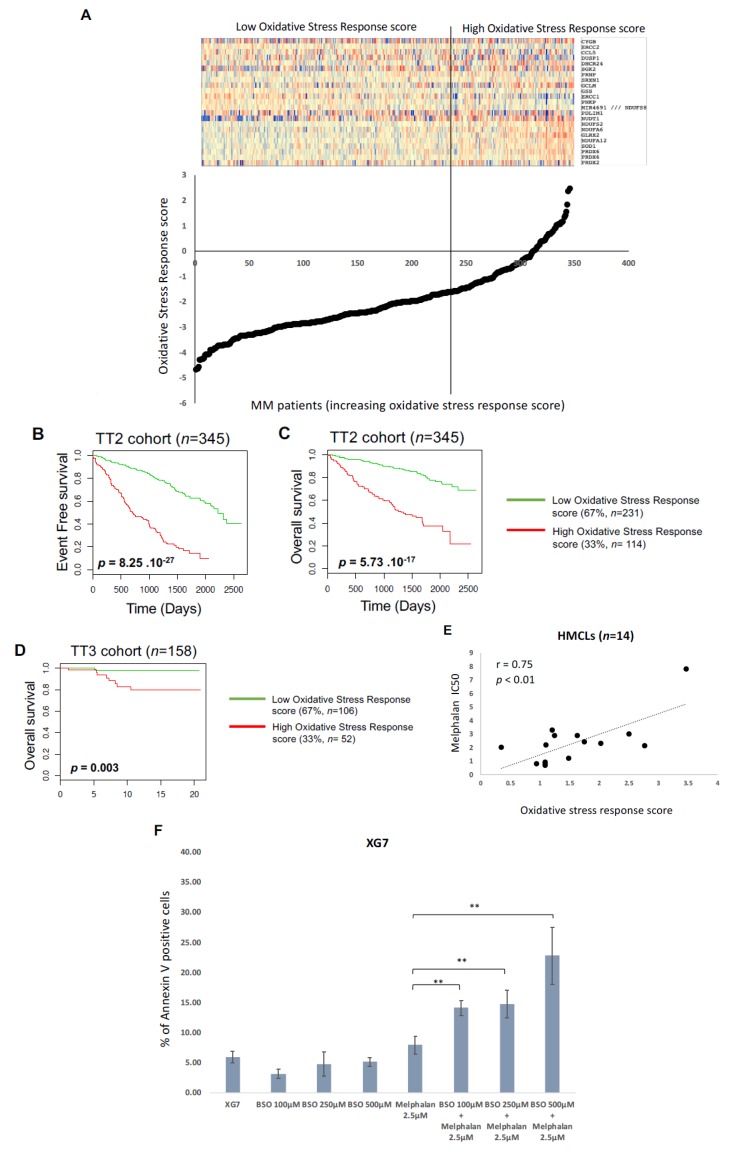

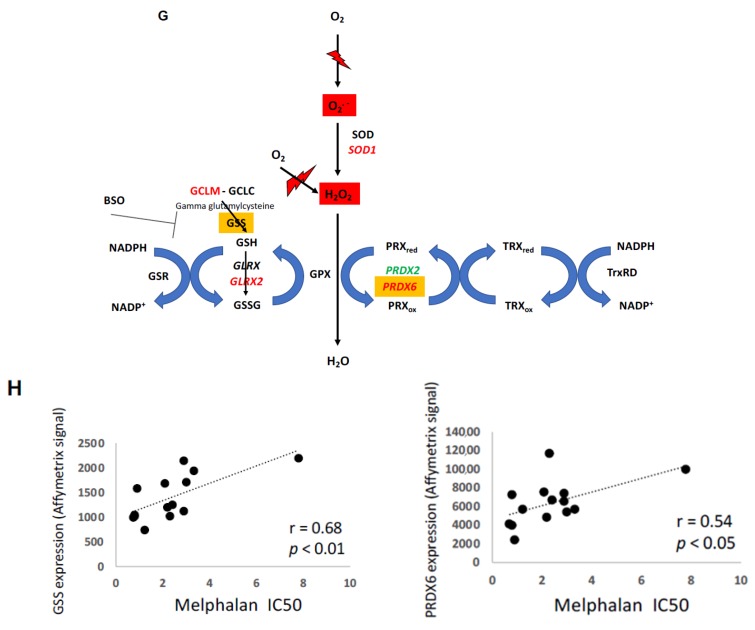
Oxidative stress related GEP-based risk score can predict for MMC resistance to melphalan. (**A**) Clustergram of oxidative stress response genes ordered from best to worst prognosis. The level of the probe set signal is displayed from low (deep blue) to high (deep red) expression. MM patients (UAMS-TT2 cohort *n* = 345) were ordered by increasing GE-based oxidative stress response score. (**B**,**C**) Patients of the UAMS-TT2 cohort (*n* = 345) were ranked according to increased oxidative stress response score and a maximum difference in EFS and OS was obtained with oxidative stress response score splitting patients into high-risk (33%) and low-risk (67%) groups. (**D**) Oxidative stress response score also had a prognostic value of an independent cohort of 158 patients from University of Arkansas for Medical Science (UAMS) treated with TT3 therapy (TT3 cohort). The parameters to compute the oxidative stress response of patients of TT3 cohort and the proportions delineating the two prognostic groups were those defined with TT2 cohort. (**E**) High level of oxidative stress response score significantly correlated with melphalan resistance in 14 HMCLs. (**F**) XG7 was treated with sublethal doses of BSO in combination with 2.5 μM of melphalan. Apoptosis induction was analyzed with Annexin V PE staining by flow cytometry. The shown data are the mean values ± SD of four separate experiments. ** *p* < 0.01 using a Student test for pairs. (**G**) Oxidative stress response in MM. Red color represents genes associated with a poor outcome in MM patients. Green color represents genes associated with a better outcome in MM patients. Yellow color represents genes significantly correlated with MMC response to melphalan. (**H**) Affymetrix gene expression level of *GSS* and *PRDX6* significantly correlated with melphalan resistance in 14 HMCLs.

A list of 46 probesets representative of genes encoding proteins involved in response to oxidative stress were selected from GSEA Gene Set RESPONSE_TO OXYDATIVE_STRESS. Using the Maxstat R function and the Benjamini Hochberg multiple testing correction, 23 genes were associated with a prognostic value in the TT2 cohort (including eight genes associated with a good prognostic value and 15 with a poor prognostic value) (Table S2). The prognostic information of these genes was gathered in a oxidative stress response score which is the sum of the beta coefficients of the Cox model for each prognostic gene, weighted by 1 according to the patient MMC signal above or below the probe set Maxstat value as previously described [33,34,35]. Using patient’s UAMS-TT2 and TT3 cohorts, the oxidative stress response score had prognostic value when splitting patients into two groups using the Maxstat R function [35]. The oxidative stress response score splits patients in a high-risk group (33%) and a low-risk group (67%) in the UAMS-TT2 and TT3 cohorts (*p* < 0.0001) (Figure 8A–D). Using our large cohort of HMCLs [23,36], we analyzed the correlation between the response of 14 HMCLs to melphalan and the oxidative stress response score. A significant correlation between oxidative stress response score and melphalan resistance was observed (*r* = 0.75; *p* < 0.01) (Figure 8E). High oxidative stress response score is associated with a poor outcome and melphalan resistance in MM. Interestingly, addition of sublethal doses of buthionine sulphoximine (BSO) that lower GSH level, results in a significant increase of melphalan toxicity in XG7 MMC (Figure 8F).

## 3. Discussion

Melphalan is widely used for the treatment of MM patients. Furthermore, despite significant advances in MM treatment, including novel agents that significantly prolonged the median survival, the majority of MM patients relapse with the development of drug resistance [37]. Minimal residual disease assessment in MM demonstrated that the persistence of malignant plasma cells in patients after high dose chemotherapy and ASCT could be detected [38,39,40,41,42]. Detection of residual malignant plasma cells, three months after high dose therapy and ASCT, is associated with a shorter progression free survival and overall survival in MM patients [38,39,40,41,42]. Furthermore, Caraux et al. demonstrated the persistence of viable malignant MM cells, within bone marrow, seven days after high dose melphalan and ASCT in two third of the patients included in the study [43]. In this study, we have confirmed that melphalan induces reactive oxygen species in MM cells and, as a consequence of this oxidative stress, oxidation of lipids and proteins and a decrease of the cellular antioxidant GSH. We also reported an activation of the NRF2 pathway after treatment with melphalan. We showed that the addition of the antioxidant GSH and, more moderately, other antioxidant compounds involved in GSH synthesis and regeneration, prevented melphalan toxicity. Other antioxidants molecules such as tiron and trolox did not protect cells from melphalan while protecting cells from bortezomib, a well-known reticulum endoplasmic stress and oxidative stress inducer [44]. We can hypothesize that melphalan and bortezomib do not induce the same ROS or that GSH protection towards melphalan is not restrained to ROS scavenging.

Surprisingly, whereas exogenous addition of GSH partially restored GSH levels in treated cells and prevented G2/M arrest, induction of ROS and cell death after treatment with melphalan, it didn’t protect cells from DNA damages induced by melphalan as demonstrated by the unchanged number of 53BP1 foci just after the onset of treatment with melphalan in the presence of GSH. These observations rule out the hypothesis of a direct drug inactivation by GSH and raise the question of the origin of cell death after treatment with melphalan as, in our cell lines and with the drug concentrations used, DNA damage is not lethal in the presence of GSH. This suggests that oxidative stress is a major cause of cell death after treatment with melphalan. Nevertheless, we can’t exclude that GSH may modulate DNA damage response and DNA repair [45,46,47]. Additionally, GSH could directly block apoptosis independently of its role on oxidative stress as it was shown for other alkylating drugs [48].

Interestingly, melphalan induced a significant decrease of the pool of intracellular GSH in MM cells. In cancer, resistance to chemotherapy have been associated to NRF2 pathway activation or elevated GSH levels [49,50]. These data underline that combination of alkylating agents with GSH-depleting agents like buthionine sulphoximine (BSO) could be of therapeutic interest in MM.

ROS include free radicals. Furthermore, considering reported ROS production in response to the effect of hypoxia [51], analyze the functions of antioxidant defense in MMC drug resistance in hypoxic conditions will be of particular interest. The reactive intermediates, produced by oxidative stress, can alter the membrane bilayers and cause the lipid peroxidation of polyunsaturated fatty acids. This lipid peroxidation with the formation of reactive compounds can lead to changes in the permeability of the membrane and can alter cell integrity [27]. Our results demonstrated that melphalan can induce protein carbonylation and lipid proxidation as a consequence of ROS induction. Recent data demonstrated an enrichment of NRF2 and endoplasmic reticulum (ER) pathways in response to alkylating agents in solid cancers [52]. Since the high level of immunoglobulin secretion requires folding in the ER lumen, the influence of NRF2 and ER homeostasis could be of interest to target MM cells [53].

We developed an oxidative stress response score based on 23 genes including 15 genes associated with a poor outcome. Interestingly, high oxidative stress response score is associated with a poor prognosis (EFS and OS) in newly diagnosed MM patients. Furthermore, a significant correlation between oxidative stress response score and melphalan resistance was observed in 14 HMCLs. Among these genes, two key determinants of GSH synthesis was identified. High expression of glutamate-cysteine ligase regulatory subunit (*GCLM*) was associated with a poor prognosis in newly diagnosed MM patients underlining the link between antioxidant defenses and resistance to treatment in MM (Figure 8G). PRDX2 and PRDX6 encoding peroxiredoxins were reported to be associated with chemoresistance in cancer [54]. PRDX6 demonstrates pro-tumorigenic functions through proliferative proliferation and invasiveness support [54]. The coper/zinc dismutase SOD1 was also identified. SOD1 is overexpressed in cancer with a major role to maintain cellular ROS under cellular critical threshold [55]. Furthermore, GSH synthetase (*GSS*) and *PRDX6* expression was significantly correlated with melphalan resistance in 14 HMCLs (Figure 8H). Interestingly, it was recently demonstrated a link between elevated ILF2 expression driven by 1q21 amplification in MM and resistance to genotoxic agents [56]. ILF2 regulates splicing transcription of DNA repair genes supporting MMC resistance to genotoxic agents. These data underline that exploiting this mechanism in combination with modulation of redox system could be of therapeutic interest to optimize the use of genotoxic agents in MM (Figure 8G).

Finally, this study shows that affecting GSH content, GSH synthesis pathways or redox status in MM cells can modulate response to treatment with melphalan.

## 4. Materials and Methods

### 4.1. Drugs and Reagents

Reduced glutathione (GSH), N-acetylcysteine (NAC), ascorbic acid (AA), vitamin E (Vit E), Tiron, Trolox, Ebselen were purchased from Sigma-Aldrich (St. Louis, MO, USA). Melphalan (Alkeran^®^) was provided by Montpellier University Hospital.

### 4.2. Human Myeloma Cell Lines Culture (HMCLs)

XG HMCLs were obtained as previously described [23,57]. LP1 cell line was purchased from DSMZ (Braunsweig, Germany). HMCLs were cultured in RPMI1640 media supplemented with glutamax (Life Technologies, Carlsbad, CA, USA), 10% fetal calf serum and recombinant IL-6 (2ng/mL) (Peprotech France, Neuilly-sur-Seine, France). HMCLs were authenticated according to their short tandem repeat profiling and their gene expression profiling using Affymetrix U133 plus 2.0 microarrays deposited in the ArrayExpress public database under accession numbers E-TABM-937 and E-TABM-1088 [23].

### 4.3. Primary Plasma Cells Culture

Bone marrow of patients presenting with previously untreated MM (*n* = 6) at the university hospital of Montpellier was obtained after patients’ written informed consent in accordance with the Declaration of Helsinki and agreement of the Montpellier University Hospital Centre for Biological Resources (DC-2008-417). Primary myeloma cells of patients were cultured with or without graded concentrations of melphalan and with or without a 5 mM GSH cotreatment. MMC cytotoxicity was evaluated using anti-CD138-phycoerythrin monoclonal antibody (Immunotech, Marseille, France) as described [33,58].

### 4.4. Cell Growth Assay

HMCLs were cultured in 96-well flat-bottom microtiter plates in RPMI1640 10% fetal calf serum (FCS) with IL-6 (2 ng/mL) for 5 days. After 24 h, cells were treated with increasing concentrations of melphalan alone or in combination with antioxidants GSH (5 mM), NAC (5 mM), AA (100 μM) or Vit E (50 μM). After 4 days of treatment, cell growth was evaluated by quantifying intracellular ATP amount with a Cell Titer Glo Luminescent Assay (Promega, Madison, WI, USA) using a Centro LB 960 luminometer (Berthold Technologies, Bad Wildbad, Germany).

### 4.5. Cell Cycle Analysis

After cell culture with melphalan alone or in combination to 5 mM GSH, cell cycle analysis was performed with the APC BrdU Flow Kit (BD Biosciences, Franklin Lake, NJ, USA) according to the manufacturer protocol and an additional step of DNA staining with 4′,6′-diamidino-2-phenylindole (DAPI) (2 µg/mL). For all flow cytometry experiments, fluorescence was quantified using a Cyan cytometer and resulting data analyzed with the Kaluza software (Beckman Coulter, Fullerton, CA, USA).

### 4.6. Caspase 3/7 and 9 Activity

Apoptosis was measured using caspase 3/7 and caspase 9 luminescence assay. XG2 and XG7 HMCLs were plated in 96-wells plate and treated with increasing concentrations of Melphalan alone or in combination with 5 mM GSH. At the end of treatment time, caspase 3/7 and caspase 9 activation was measured with Caspase Glo Assay (Promega) according to manufacturer’s protocol and after normalization with ATP content. For Caspase Glo 3/7 and Caspase Glo 9 assays, the intensity of bioluminescence is proportional to the activation of caspases 3/7 and caspase 9 respectively.

### 4.7. Gene Expression Profiling and Statistical Analyses

Gene expression data were normalized with the MAS5 algorithm and analyses processed with GenomicScape (http://www.genomicscape.com) [59] the R.2.10.1 and bioconductor version 2.5 programs [60]. Gene Set Expression Analysis Univariate and multivariate analysis of genes prognostic for patients’ survival was performed using the Cox proportional hazard model. Difference in event free survival between groups of patients was assayed using Maxstat algorithm [35] and survival curves plotted using the Kaplan–Meier method.

### 4.8. DNA Repair Foci Quantification

After deposition on slides using a Cytospin centrifuge (Thermo Fisher Scientific, Waltham, MA, USA), cells were fixed with 4% PFA, permeabilized with 0.5% Triton in PBS and saturated with 5% bovine milk in PBS. The rabbit anti-53BP1 antibody (clone NB100-304, Novus Biologicals, Cambridge, UK) was diluted 1/300 and deposited on cytospins for 90 min at room temperature. Slides were washed twice and secondary anti rabbit IgG conjugated to alexa 488 (diluted 1/500; Invitrogen, Life Technologies) was added for 45 min at room temperature. Slides were washed and mounted with Vectashield and 1% DAPI. Images and fluorescence were captured with an Axio Imager Z2 microscope (×63 objective, Zeiss, Oberkochen, Germany), analyzed with Metafer (version 3.6, Altlussheeim, Germany) and ImageJ softwares (National Institutes of Health, Bethesda, MD, USA). The number of 53BP1 and γH2AX foci was counted in at least 300 nuclei.

### 4.9. Detection of Intracellular Reactive Oxygen Species (ROS)

ROS were quantified using CM-H2DCFDA (Molecular Probes, Eugene, OR, USA) following the manufacturer’s protocol. Briefly, cells were incubated in pre-warmed PBS with 6 µM of the probe for 20 min in the dark at 37 °C. Cells were then centrifuged and resuspended in growth medium for 2 h at 37 °C before fluorescence detection by flow cytometry analysis.

### 4.10. Reduced Glutathione Quantification

Reduced GSH was measured using a colorimetric Gluathione Assay kit from Abnova (Walnut, CA, USA) according to manufacturer’s instructions. Briefly, DTNB and glutathione (GSH) react to generate GSSG and 2-nitro-5-thiobenzoic acid which has yellow color. Therefore, reduced GSH concentration can be determined by measuring absorbance at 412 nm (Beckman Coulter).

### 4.11. Protein Carbonyl Oxidation Assay

The most common products of protein oxidation in biological samples are the protein carbonyl derivatives. The quantification of protein carbonyl was performed using the OxiSelect Protein Carbonyl ELISA Kit (STA-310, Cell Biolabs, Inc., San diego, CA, USA) according to manufacturer’s instructions.

### 4.12. Malondialdehyde (MDA) Quantification Assay

Thiobarbituric Acid Reactive Substances (TBARS) has been used for MDA assay for screening and monitoring lipid peroxidation. MDA quantification was performed using OxiSelectTM TBARS Assay Kit (STA-310, Cell Biolabs, Inc.) according to manufacturer’s instructions.

### 4.13. Study of Apoptosis

HMCLs were cultured in 24-well, flat-bottomed microtiter plates at 10^5^ cells per well in RPMI1640–10% FCS culture medium, IL-6 (3 ng/mL) and with or without BSO (Sigma), and melphalan. After 4 days of culture, cells were washed twice in PBS and apoptosis was assayed with PE-conjugated Annexin V labeling (BD Biosciences, San Jose, CA, USA) using a Fortessa flow cytometer (BD).

### 4.14. Statistical Analysis

Statistical differences were tested using Student *t*-test or Wilcoxon test.

### 4.15. Western Blot Analysis

Cells were lysed in 10 mM Tris-HCl (pH 7.05), 50 mM NaCl, 50 mM NaF, 30 mM sodium pyrophosphate, 1% Triton X-100, 5 μM ZnCl_2_, 100 μM Na_3_VO4, 1 mM dithiothreitol, 20 mM β-glycerophosphate, 20 mM p-nitrophenol phosphate, 20 μg/mL aprotinin, 2.5 μg/mL leupeptin, 0.5 mM phenylmethylsulfonyl fluoride, 0.5 mM benzamidine, 5 μg/mL pepstatin and 50 nM okadaic acid. Lysates were resolved on 12% sodium dodecyl sulfate-polyacrylamide gel electrophoresis and transferred to a nitrocellulose membrane (Schleicher and Schuell, Kassel, Germany). Membranes were blocked for 2 h at room temperature in 140 mM NaCl, 3 mM KCl, 25 mM Tris-HCl (pH 7.4), 0.1% Tween 20 (tris-buffered saline Tween-20), 5% non-fat milk, and then immunoblotted with monoclonal mouse anti-γ-GCSm antibody (Santa Cruz Biotechnology, Heidelberg, Germany), polyclonal goat-anti-γ-GCSc antibody (C-15, Santa Cruz Biotechnology), monoclonal rabbit anti-HO-1 antibody (Cell Signaling Technology, Beverly, MA, USA) and monoclonal mouse anti-NQO1 antibody (Cell Signaling Technology). To control protein loading, β-actin amounts were probed with a mouse monoclonal anti- β-actin antibody (Sigma). Primary antibodies were visualized with peroxidase-conjugated antibodies by an enhanced chemiluminescence detection system. Blot densitometry was quantified using the NIH ImageJ software (National Institutes of Health, Bethesda, MD, USA) and protein expression normalized to β-actin levels.

## 5. Conclusions

Targeting GSH synthesis and regeneration pathways as NRF2 pathway is a raising new therapeutic strategy for oxidative stress associated diseases or to increase the efficiency of drugs inducing oxidative imbalance. Various molecules and inhibitors are currently in clinical trial or FDA approved for the treatment of promyelocytic leukemia, lung cancer, breast cancer, ovarian carcinoma, leukemia, lymphoma [61,62,63]. Interestingly sublethal doses of BSO combined with melphalan demonstrated a synergy. Modulation of redox system such as inhibition of GSH synthesis and regeneration or NRF2 pathway inhibition could therefore represent appealing new therapeutic strategies to overcome melphalan resistance in multiple myeloma.

## Supplementary Materials

The following are available online at https://www.mdpi.com/2072-6694/11/4/439/s1, Figure S1: Relative intracellular GSH content in XG7 and XG2 myeloma cells, Figure S2: Kinetics of melphalan effect on the viability and cell cycle of XG7 and XG2 myeloma Cells, Figure S3: Kinetics of melphalan effect on the viability and cell cycle of XG2 myeloma cells, Figure S4: Pretreatment with GSH doesn’t prevent the induction of 53BP1 foci after treatment of XG2 HMCL with melphalan, Figure S5: XG2 HMCL was cultured for 4 days in 96-well flat-bottom microtitre plates in RPMI 1640 medium, 10% fetal calf serum, 2 ng/ml IL-6 culture medium (control) and graded concentration of Melphalan with or without GSH. Data are mean values of three experiments determined on sextuplet culture wells, Figure S6: Addition of 5 mM of GSH protects MM cells from Melphalan induced toxicity, Table S1: XG2 cells were treated with 5 μM melphalan with or without cotreatment with 5 mM GSH. Four days after treatment viability was determined with trypan blue exclusion assay. Cell count and percentage of viable cells for 4 independent experiments are shown in the table, Table S2: Oxidative stress response score genes.
