# Supplementary material for: Antioxidant Defenses Confer Resistance to High Dose Melphalan in Multiple Myeloma Cells
Source: Cancers (Basel). 2019 Mar 28;11(4):439. doi: 10.3390/cancers11040439 (PMC6521290; doi:10.3390/cancers11040439)
Supplement: Supplementary file 1 [file cancers-11-00439-s001.pdf]

Supplementary Materials: Antioxidant Defenses Confer Resistance to High Dose Melphalan in Multiple Myeloma Cells

Claire Gourzones, Céline Bellanger, Sylvain Lamure, Ouissem Karmous Gadacha, Elvira Garcia De Paco, Laure Vincent, Guillaume Cartron, Bernard Klein and Jérôme Moreaux

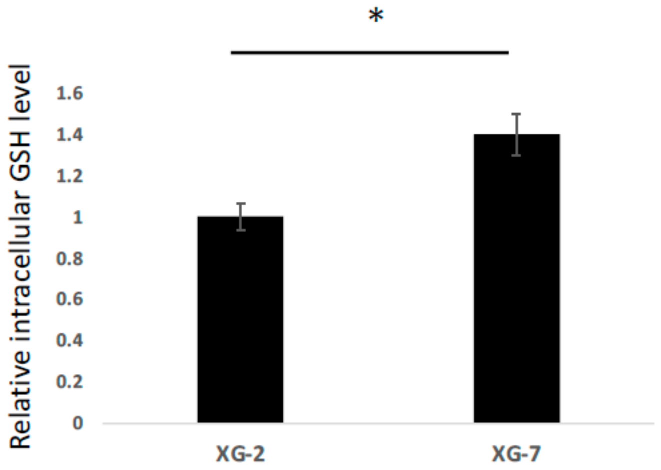

Figure S1. Relative intracellular GSH content in XG7 and XG2 myeloma cells. Intracellular GSH level was measured in accordance with Glutathione Assay Kit protocol (ABNOVA). Data are represented as the average +/- SD of an experiment representative of three performed in triplicate.

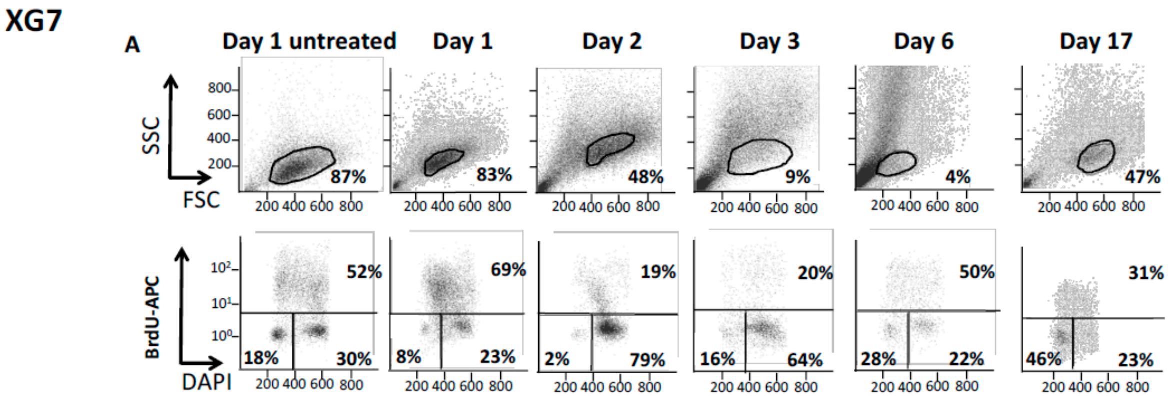

B

Treatment with 15 µM melphalan	-	+	+	+	+	+
Day post-treatment	1	1	2	3	6	17
Viable cells (Cumulative count)	390 000	366 000	342 000	251 000	63 000	5 000 000
% viable cells	87	83	48	9	4	47
GO-G1% total gated cells	18	8	2	16	28	46
S % Total gated cells	52	69	19	20	50	31
G2 % total gated cells	30	23	79	64	22	23

XG2

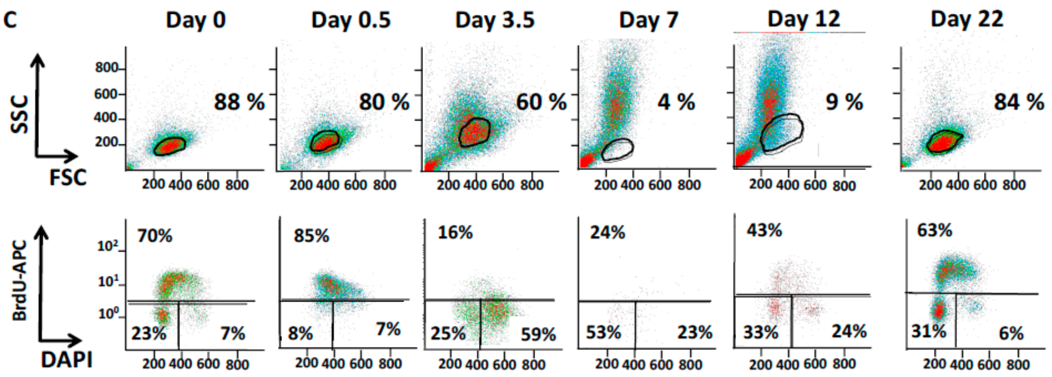

D

Day post-treatment	0	0.5	3.5	7	12	22
Viable cells	216 000	162 000	72 000	23 000	135 000	1 300 000
% viable cells	88	80	60	4	9	84
G0-G1 % total gated cells	23	8	25	53	33	31
S % total gated cells	70	85	16	24	43	63
G2 % total gated cells	7	7	59	23	24	6

Figure S2. Kinetics of melphalan effect on the viability and cell cycle of XG7 and XG2 myeloma cells. **A:** XG7 cells were treated with 15 µM melphalan (i.e., IC90) and FSC/SSC and cell cycle analyses were performed by flow cytometry after BrdU/DAPI staining before treatment (day 0) and at day 1, 2, 3, 6, 17 after the onset of treatment. **B**: The percentage of cells in each phase of the cell cycle is indicated. The percentage of viable cells was determined using FSC/SSC characteristics and cell count. **C:** XG2 cells were treated with 5 µM melphalan (i.e., IC97) and FSC/SSC and cell cycle analysis were performed by flow cytometry after BrdU/DAPI staining before treatment (day 0) and at day 0.5, 3.5, 7, 12, 22 after the onset of treatment. **D:** The percentage of cells in each phase of the cell cycle is indicated. The percentage of viable cells was determined using FSC/SSC characteristics and cell count.

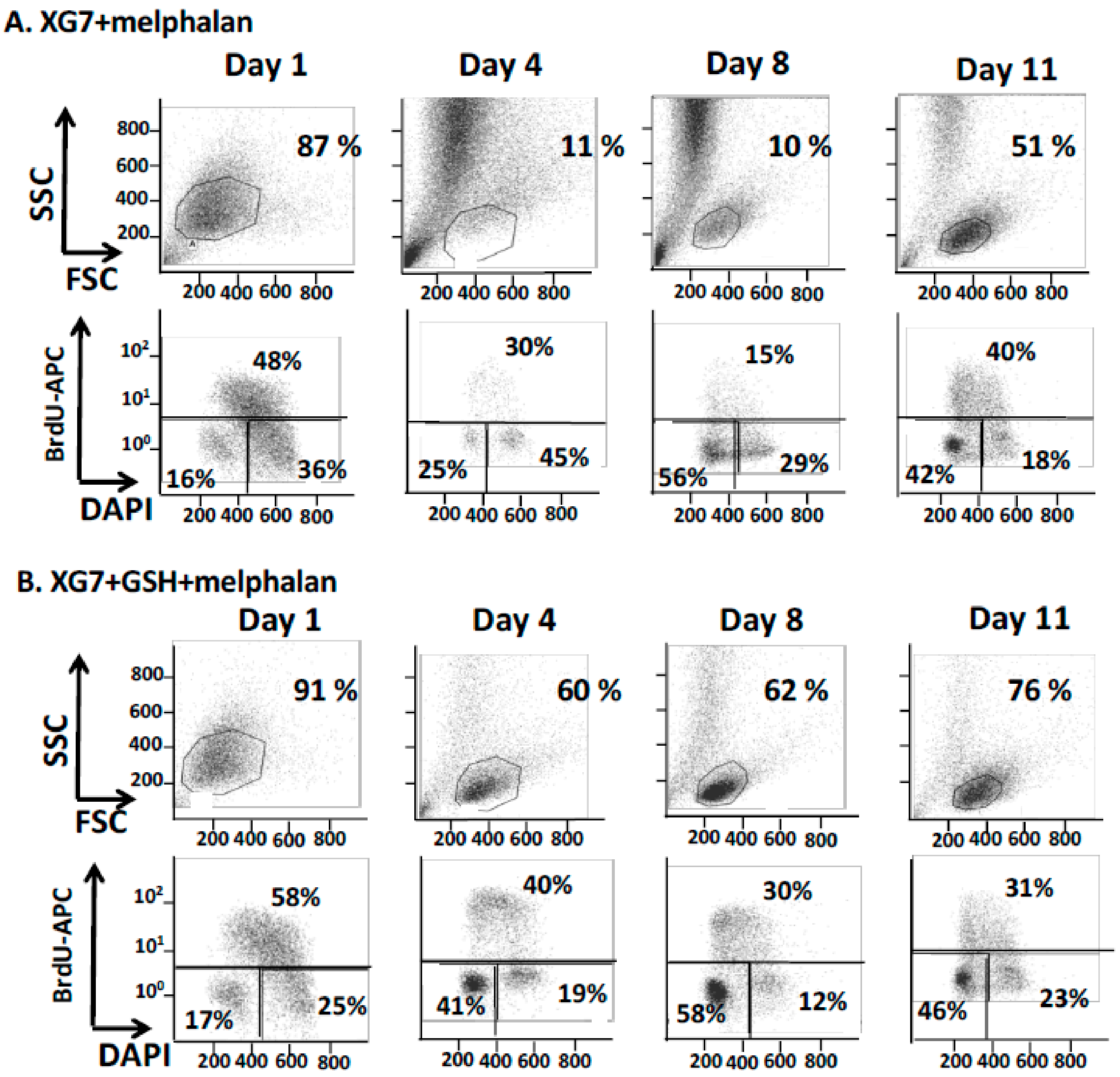

Figure S3. Kinetics of melphalan effect on the viability and cell cycle of XG7 myeloma cells. (**A**) XG7 cells were treated with 15 µM melphalan and FSC/SSC and cell cycle analyses were performed by flow cytometry, using BrdU incorporation and labeling with an anti-BrdU antibody and DAPI, before treatment (day 0) and at day 1, 4, 8, 11 after the onset of treatment. (**B**) The percentage of cells in each phase of the cell cycle is indicated. The percentage of viable cells was determined using FSC/SSC characteristics and cell count.

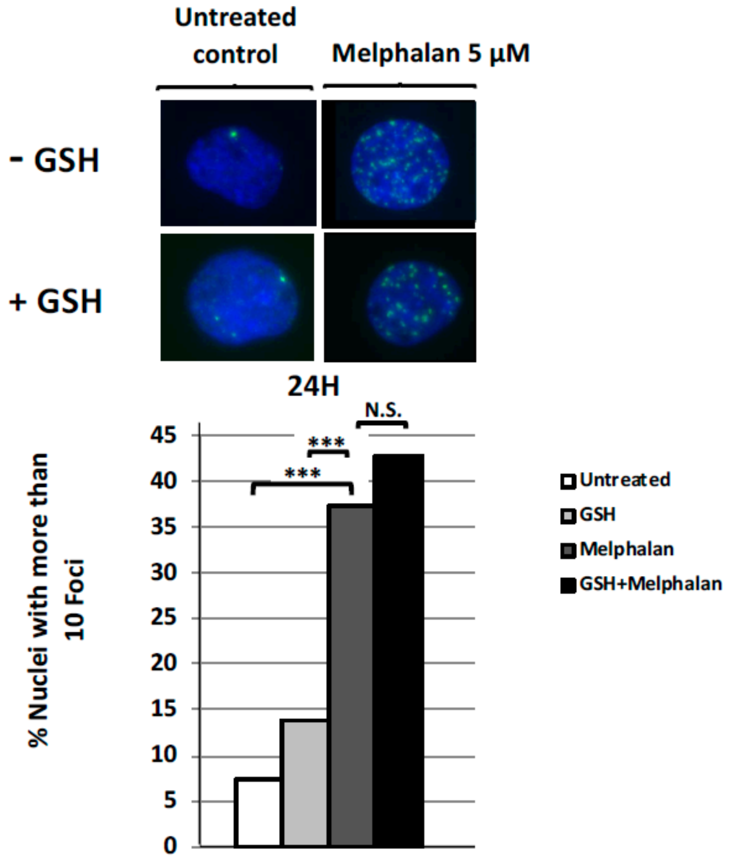

Figure S4. Pretreatment with GSH doesn't prevent the induction of 53BP1 foci after treatment of XG2 HMCL with melphalan. XG2 cells were treated with 5 µM of melphalan with or without a pretreatment with 5 mM GSH. Cells were harvested after 24h and 53BP1 foci detected using immunofluorescence. A representative image for each condition and the number of foci per nuclei are shown on the figure. *** $p < 0.001$.

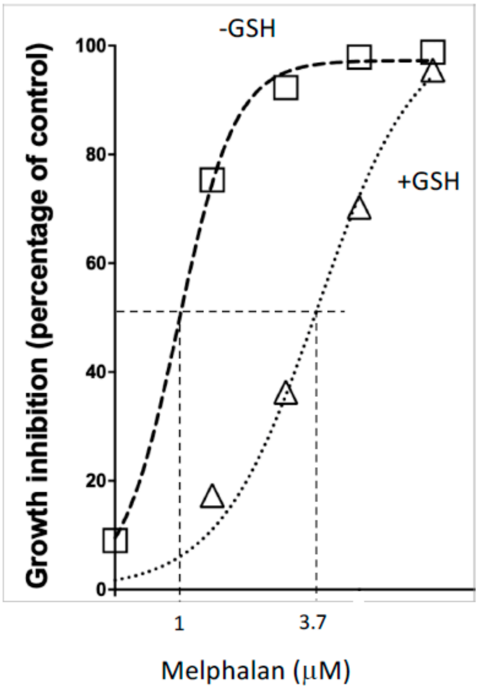

Figure S5. XG2 HMCL was cultured for 4 days in 96-well flat-bottom microtitre plates in RPMI 1640 medium, 10% fetal calf serum, 2 ng/ml IL-6 culture medium (control) and graded concentration of

Melphalan with or without GSH. Data are mean values of three experiments determined on sextuplet culture wells.

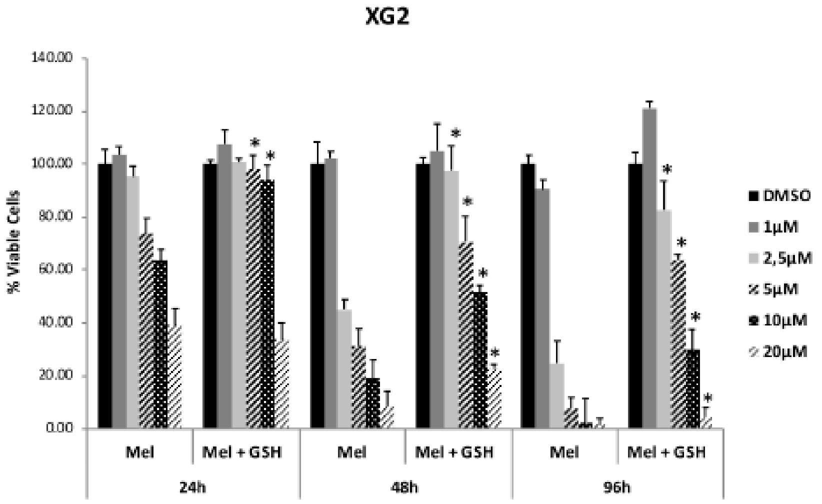

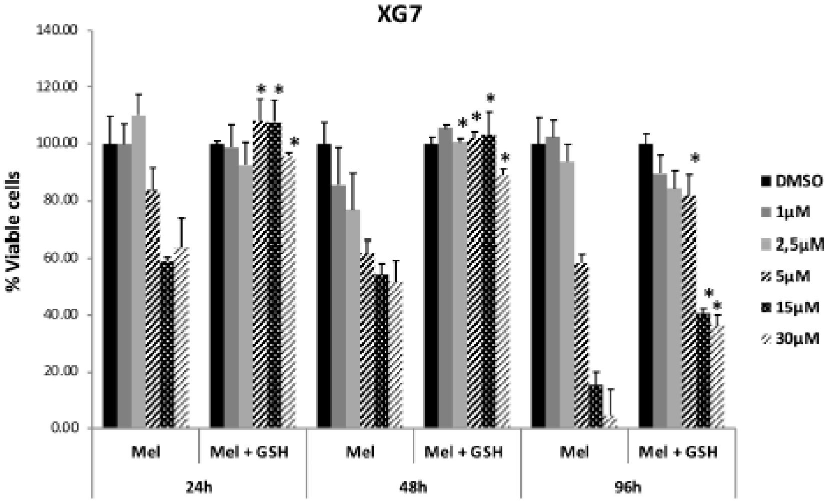

Figure S6. Addition of 5 mM of GSH protects MM cells from Melphalan induced toxicity. XG2 and XG7 HMCLs were cultured for 1, 2 and 4 days in 96-well flat-bottom microtitre plates in RPMI 1640 medium, 10% fetal calf serum, 2 ng/ml IL-6 culture medium (control) and graded concentration of Melphalan with or without GSH. Data are mean values ± SD of three experiments determined on sextuplet culture wells. * $p < 0.05$ compared to melphalan alone.

Table S1. XG2 cells were treated with 5 µM melphalan with or without cotreatment with 5 mM GSH. Four days after treatment viability was determined with trypan blue exclusion assay. Cell count and percentage of viable cells for 4 independent experiments are shown in the table.

Treatment	CELL COUNT ± sd	VIABILITY % ± sd
Untreated	1 685 000 ± 574	739 93 ± 4
GSH 5 mM	1 556 667 ± 592	687 95 ± 2
Melphalan 5 µM	61 208 ± 18	244 18 ± 8
GSH + Melphalan	476 667 ± 148	163 73 ± 13

Table S2. Oxidative stress response score genes.

Probeset	Name	Multiple Testing Corrected *p* Value	Hazard_Ratio	Prognostic
204655_at	CCL5	0.04	0.64	Good
1553572_a_at	CYGB	0.02	1.6	Bad
200862_at	DHCR24	0.02	1.7	Bad
201044_x_at	DUSP1	0.02	0.58	Good
203720_s_at	ERCC1	0.01	0.58	Good
235399_at	ERCC2	0.009	0.53	Good
236140_at	GCLM	0.02	1.8	Bad
219933_at	GLRX2	0.0003	2.4	Bad
211630_s_at	GSS	0.03	0.67	Good
203190_at	MIR4691///NDUFS8	0.02	0.63	Good
223244_s_at	NDUFA12	0.01	1.7	Bad
202001_s_at	NDUFA6	0.03	1.5	Bad
201966_at	NDUFS2	0.00003	2.4	Bad
204766_s_at	NUDT1	0.03	1.9	Bad
208690_s_at	PDLIM1	0.03	1.7	Bad
218961_s_at	PNKP	0.02	0.64	Good
211658_at	PRDX2	0.03	0.65	Good
200844_s_at	PRDX6	0.009	1.7	Bad
200845_s_at	PRDX6	0.02	1.9	Bad
215707_s_at	PRNP	0.03	1.5	Bad
230573_at	SGK2	0.04	1.6	Bad
200642_at	SOD1	0.0217	1.6	Bad
225252_at	SRXN1	0.01066	1.8	Bad

© 2019 by the authors. Licensee MDPI, Basel, Switzerland. This article is an open access article distributed under the terms and conditions of the Creative Commons Attribution (CC BY) license (http://creativecommons.org/licenses/by/4.0/).
